# Using Carbomer-Based Hydrogels for Control the Release Rate of Diclofenac Sodium: Preparation and In Vitro Evaluation

**DOI:** 10.3390/ph13110399

**Published:** 2020-11-17

**Authors:** Muhammad Suhail, Pao-Chu Wu, Muhammad Usman Minhas

**Affiliations:** 1 School of Pharmacy, Kaohsiung Medical University, 100 Shih-Chuan 1st Road, Kaohsiung City 80708, Taiwan; Suhailpharmacist26@gmail.com; 2 Department of Medical Research, Kaohsiung Medical University Hospital, Kaohsiung 80708, Taiwan; 3 Drug development and value creation research center, Kaohsiung Medical University, Kaohsiung 80708, Taiwan; 4 College of Pharmacy, University of Sargodha, Sargodha 40100, Pakistan

**Keywords:** hydrogels, carbopol, diclofenac sodium, in-vitro study

## Abstract

The aim of the current research work was to prepare Car934-g-poly(acrylic acid) hydrogels by the free-radical polymerization technique. Various concentrations of carbopol, acrylic acid and ethylene glycol dimethacrylate were employed for the fabrication of Car934-g-poly(acrylic acid) hydrogels. Fourier-transform infrared spectroscopy (FTIR), Thermogravimetric analysis (TGA), Differential scanning calorimetry (DSC), Scanning electron microscope (SEM) and Powder X-ray diffractometry (PXRD) studies were performed to know the structural arrangement, thermal stability, physical appearance and amorphous network of developed hydrogels. FTIR analysis revealed that carbopol reacted with acrylic acid during the process of polymerization and confirmed the grafting of acrylic acid over the backbone of carbopol. TGA and DSC studies showed that developed hydrogels were thermally stable. Surface morphology was analyzed by SEM, which confirmed a porous network of hydrogels. PXRD analysis indicated that crystallinity of the drug was reduced by the amorphous network of hydrogels. Furthermore, swelling studies for all developed hydrogels were performed at both media, i.e., pH 1.2 and 7.4, and higher swelling was exhibited at pH 7.4. Sol–gel analysis was performed to evaluate the soluble unreacted part of the fabricated hydrogels. Similarly, an in-vitro study was conducted for all hydrogel formulations at both acidic (pH 1.2) and basic (pH 7.4) mediums, and a greater drug release was observed at pH 7.4. Different kinetics such as zero-order, first-order, the Higuchi model and the Korsmeyer–Peppas model were applied to know the mechanism of release order of drugs from the hydrogels.

## 1. Introduction

Hydrogels are three-dimensional structures with the capability to absorb and hold a high quantity of water without losing structural consistency [1]. Hydrogels are very stable by nature; due to which, the solutions absorbed by hydrogels remain inside its network, even in the presence of any external force [2]. Due to the presence of a high number of hydrophilic groups like –OH, –SO3H, –COOH, –NH2, etc. on a polymer chain, a huge quantity of water is observed by hydrogels [3,4]. Similarly, a significant role is played by these different hydrophilic groups of polymers in the formation of noncovalent bonds of hydrogels with other numerous biological tissues like epithelial tissues and mucous membranes [5]. Two types of crosslinking occur in hydrogels, i.e., (a) physical or (b) chemical, which restricts the hydrogels from being dissolved even when holding a high concentration of water or other fluids [6]. In physical hydrogels, crosslinking arises due to noncovalent bonding such as hydrogen bonding amid the polar groups on the chains of the polymer, while, in chemical hydrogels, crosslinking develops through covalent bonds among various functional groups on the chains of the polymer enabled through distinct crosslinking agents [7]. While having distinct properties, hydrogels are considered as a potential candidate for various biomedical applications, including drug delivery and tissue engineering, due to their super-absorbency, softness, viscoelasticity, hydrophilicity, biocompatibility and biodegradability. Prominently, minor damage to the tissue or minor toxicity is caused by hydrogels. The reversible responses to various stimuli such as pH, temperature, electric field, magnetic field, biological molecules and ionic strength of a solution is another astonishing property of hydrogels [8,9] that enhances their importance further, particularly for widespread biomedical applications [10].

Stimuli-sensitive hydrogels are a special type of hydrogel that are very sensitive by nature to certain ecological changes, and responses are shown by either altering their volume or shape once visible to a specific condition. The external stimulus may be physical, chemical or biological. The physical stimuli include pressure, light, temperature, ultrasound, electric field and magnetic field, while chemical stimuli are redox, ionic strength, pH, glucose and CO_2_, whereas biological stimuli are antigens, glutathione, enzymes and DNA [11]. The most-studied hydrogels amid stimuli-responsive hydrogels are pH-sensitive hydrogels. The rapid changes that are exhibited by stimuli-sensitive hydrogels are shrinkage and swelling following exposure to a particular stimulus, leading to the transition of the volume phase. The response rate of these types of hydrogels depends upon the shape, size, crosslinking bulk, composition and number of ionic groups and is enhanced via an increase in the ionic group number and pore size, as well as by reduction of their density of crosslinking and size [12].

Carbopol polymers prepare stimuli-responsive hydrogels that bring about changes in swelling behavior when exposed to external stimuli such as temperature, pH, light or electric field [13,14,15]. Carbopol is also known as smart gels or environmentally responsive polymers [16,17]. Currently, carbopol is considered as a suitable candidate for the preparation of different types of polymeric systems, especially for controlled drug-delivery systems, and plays an important role in drug delivery to a specific area of the body. In pH-sensitive hydrogel networks, carbopol delivers the maximum drug in an alkaline medium because of its greater swelling at alkaline pH [18]. Acrylic acid is a soluble polymer that has attracted attention because of its widespread biomedical and pharmaceutical applications [19,20]. Acrylic acid is a pH-sensitive polymer used in stimuli-sensitive polymeric carrier systems, especially in pH-sensitive hydrogels like carbopol, and has maximum swelling at alkaline pH and, hence, releases the drug in a high concentration at alkaline pH [21].

Diclofenac sodium (DS) is prescribed mostly as a nonsteroidal anti-inflammatory drug (NSAID) for inflammation and pain, acting as a modest, competitive and irreversible inhibitor of the enzyme prostasin synthase [22]. Inhibition of the cyclooxygenase-2 (COX-2) enzyme with a higher potency as compared to COX-1 is the one of the key benefits of diclofenac derivatives as compared to other conventional NSAIDs. Besides efficient activity, DS has some disadvantages, such as its rapid metabolism due to a short half-life, high protein binding and a very high pre-systemic metabolism. These all generate the need for frequent high doses of DS, which further causes severe side effects like cardiac, gastrointestinal, hepatic and renal adverse effects [23]. In order to overcome all these limitations, Carbopol934-g-poly(acrylic acid) hydrogels were prepared to prolong the release of diclofenac sodium in a controlled way. Different formulations with varying concentrations of constituents were assessed and their various parameters evaluated. Different characterizations like Scanning electron microscope (SEM), Fourier-transform infrared spectroscopy (FTIR), Thermogravimetric analysis(TGA), Differential scanning calorimetry (DSC), and Powder X-ray diffractometry (PXRD) were performed in order to know the various features of the developed hydrogels, while the swelling behavior of the developed hydrogels was analyzed at various pH media concentrations systematically. This work also emphasized in-vitro drug release and kinetic modeling to analyze the percent drug release and order release mechanism of the drug from the fabricated hydrogels.

## 2. Results and Discussion

### 2.1. Analysis of FTIR

**Figure 1:**
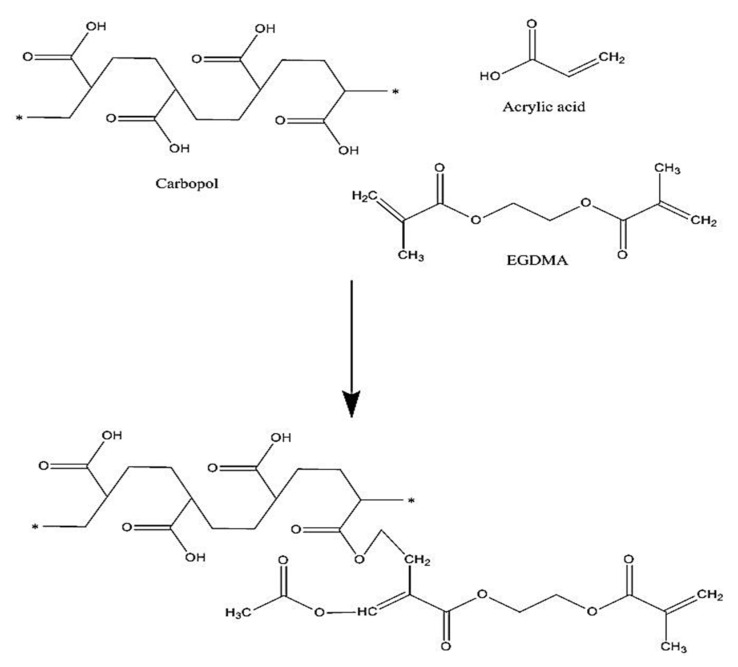
Proposed chemical structure of Car934-g-poly(acrylic acid) hydrogels.

**Figure 2:**
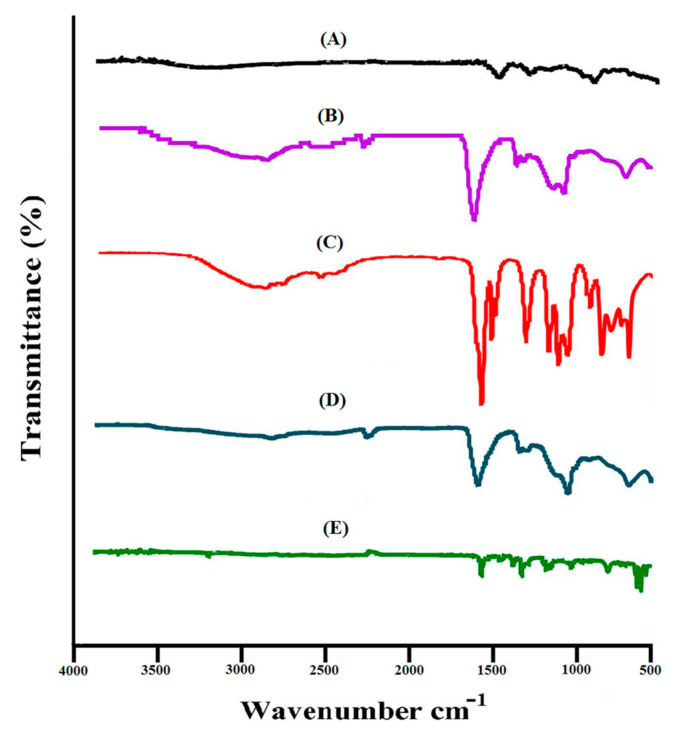
FTIR spectra of (**A**) DS, (**B**) carbopol, (**C**) AA, (**D**) unloaded Car934-g-poly(acrylic acid) hydrogel and (**E**) loaded Car934-g-poly(acrylic acid) hydrogel.

The proposed chemical structure of Car934-g-poly (acrylic acid) hydrogel is shown in Figure 1. The spectra of FTIR for DS, carbopol, acrylic acid (AA), unloaded Car934-g-poly (acrylic acid) hydrogels and drug-loaded Car934-g-poly(acrylic acid) hydrogels are shown in Figure 2A–E, correspondingly. FTIR spectrum of DS (Figure 2A) indicates COOH-stretching vibrations at the peak 3340 cm^−1^, while at peaks 1650 and 3480 cm^−1^, C=C and N–H stretching are observed, correspondingly [24]. Swain et al. (2015) reported the above peaks in his studies in the same range as in our studies [25].

Likewise, the FTIR spectrum of carbopol (Figure 2B) indicates a stretching vibration of R-CH2 (alkyl group), OH (hydroxyl group) and C=O at the characteristic peaks 3010, 2650 and 1690 cm^−1^, respectively [24,26,27]. Prominent peaks of AA (Figure 2C) indicate the stretching vibration of –CH2 and –C–C at 2945 and 1560 cm^−1^, whereas the stretching vibration of –C=O and –C–O–C assigns prominent bands at 1250 and 1180 cm^−1^ [28].

The FTIR spectra of unloaded Car934-g-poly (acrylic acid) hydrogels (Figure 2D) specify a shift in position of bands for different functional groups of carbopol and AA due to the electrostatic interactions between them. The characteristic bands of carbopol at 3010 and 2650 cm^−1^ are shifted to the 2950 and 2460 cm^−1^ peaks of unloaded Car934-g-poly (acrylic acid) hydrogels. Similarly, 1560 and 1250 cm^−1^ bands of AA are modified to 1605 and 1330 cm^−1^, respectively. Some peaks disappear, while new peaks are formed. This change in the intensity of the carbopol and AA peaks indicates the development of a new hydrogel network where the monomer is successfully grafted over the polymer backbone. Similarly, as shown in Figure 2E, two characteristics peaks of the drug at 3340 and 3480 cm^−1^ are shifted to 2270 and 3250 cm^−1^ in loaded Car934-g-poly(acrylic acid) hydrogels due to the stretching vibration of the COOH and N–H functional groups. This all indicates that the drug is completely loaded by the hydrogel network, and no interaction is formed between the drug and hydrogel contents [29].

### 2.2. Thermal Analysis

**Figure 3:**
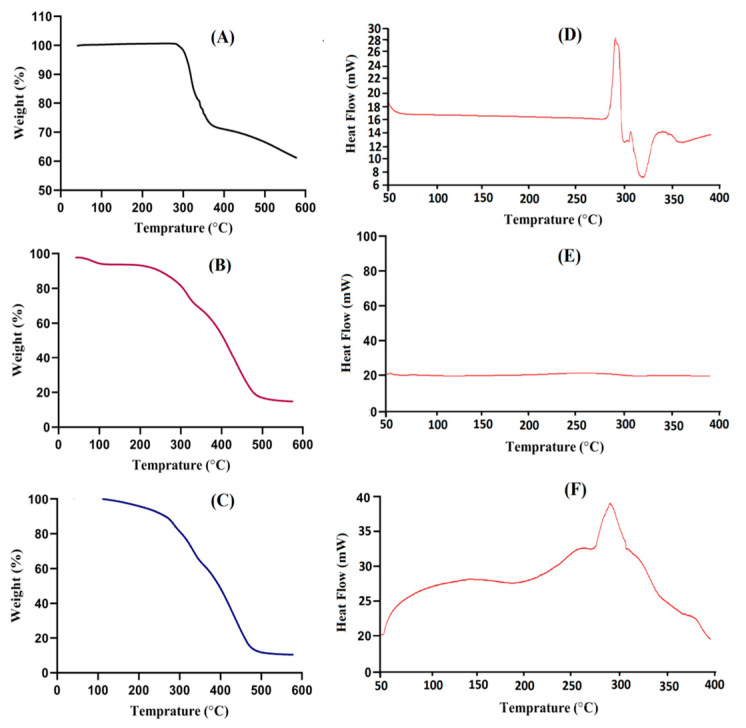
TGA of (**A**) DS, (**B**) carbopol and (**C**) Car934-g-poly(acrylic acid) hydrogel. DSC of (**D**) DS, (**E**) carbopol and (**F**) Car934-g-poly(acrylic acid) hydrogel.

As shown in Figure 3A, a TGA of DS shows a loss of weight at three different steps. At the first step, a 23% loss of weight is observed at the temperature range of 315–360 °C, followed by dehydration. At the second step, a weight loss of 17% is observed as the temperature reaches 460 °C. The final step is the pyrolysis of the drug, initiating from 460 °C until entirely paralyzed [30]. Similarly, the TGA of carbopol (Figure 3B) assigns the weight loss at three different steps. Initially, a 10% weight loss is observed at the temperature of 100 °C, followed by a loss of moisture; after this, as the temperature rises and reaches 320 °C, a weight loss of 23% is detected because of decarboxylation, the development of unsaturated structures and the polymer’s depolymerization; finally, carbopol degradation starts from 400 °C until completely paralyzed [31].

As compared to the drug and carbopol, the stability of the developed hydrogel network is higher, as shown in Figure 3C. It is clear from the thermogram of the developed hydrogels that the degradation half-life of the developed hydrogels (t1/2 = 500 °C) is higher than the degradation lives of DS and carbopol, i.e., DS (t1/2 = 360 °C) and carbopol (t1/2 = 320 °C), respectively. The initial weight loss of Car934-g-poly(acrylic acid) hydrogels is 5% at 250 °C, followed by a weight loss of 25% as the temperature reaches 370 °C and, finally, the weight loss of developed hydrogels starts from 500 °C up to its entire degradation. The enhancement in thermal stability of the developed hydrogel can be endorsed to crosslinking and grafting reactions [32]. 

The DSC of DS, carbopol and the Car934-g-poly (acrylic acid) hydrogel is shown in Figure 3D–F. The DSC of DS (Figure 3D) indicates two endothermic peaks at 285 °C and 325 °C, respectively. Likewise, at 295 °C and 347 °C, two exothermic peaks are observed, where the first peak indicates the glass transition temperature and the second peak assigns the drug degradation. Shen, X. et al. (2011) found an endothermic peak of DS at 285 °C [33].

The DSC of carbopol is shown in Figure 3E. Two endothermic peaks are observed at 65 °C and 232 °C. The first endothermic peak may be due to the evaporation of unbound water present in the polymer backbone, while the latter peak may be assigned due to the formation of anhydrides in carbopol [34]. Likewise, two exothermic peaks are observed at 93 °C and 225 °C [35], respectively. 

The DSC of Car934-g-poly (acrylic acid) hydrogels (Figure 3F) indicates two endothermic peaks at about 155 °C and 255 °C, followed by a loss of moisture of the polymer. Similarly, two endothermic peaks are indicated at 280 °C and 310 °C, respectively, followed by a phase glass transition. This indicates that developed hydrogels lead to thermal degradation in the range of 350–400 °C, although the thermal stability of the developed hydrogels exhibits better stability than the individual components [36].

### 2.3. Scanning of Electron Microscopy (SEM) Analysis

**Figure 4:**
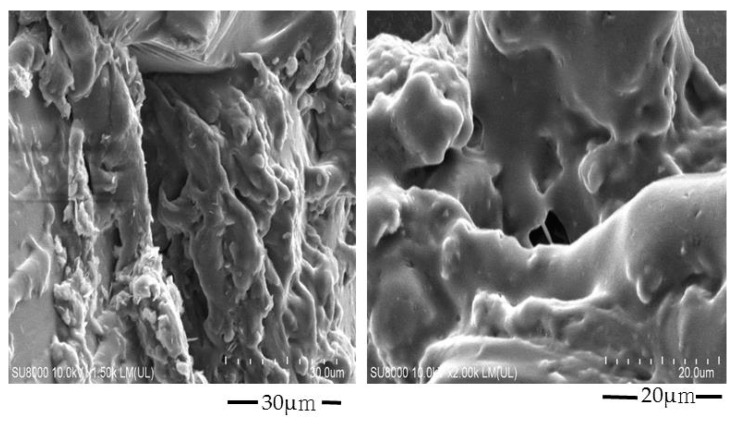
Scanning electron microscopy of the Car934-g-poly(acrylic acid) hydrogels.

The surface morphology of the Car934-g-poly (acrylic acid) hydrogels was carried out by scanning electron microscopy. A sporous structure is seen in Figure 4, which determines the high swelling of the developed hydrogels. The greater the porosity, the higher the amount of water penetrating the hydrogel network and, thus, the higher the swelling of the hydrogels [37].

### 2.4. Powder X-Ray Diffractometry (PXRD) Analysis

**Figure 5:**
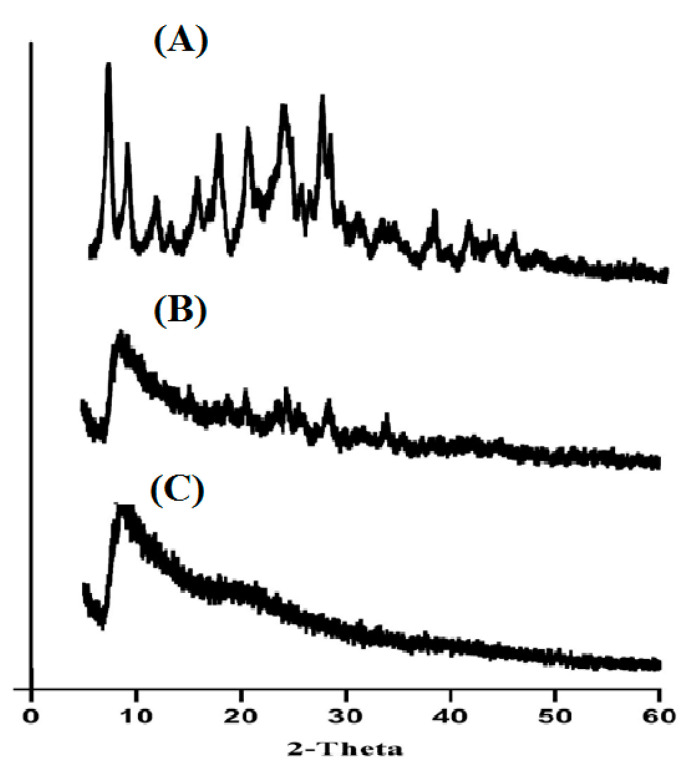
XRD of (**A**) DS, (**B**) unloaded Car934-g-poly(acrylic acid) hydrogel and (**C**) loaded Car934-g-poly(acrylic acid) hydrogel.

In Figure 5A–C, a PXRD study of DS, drug-free Car934-g-poly (acrylic acid) hydrogels and drug-loaded Car934-g-poly (acrylic acid) hydrogels is shown. Different peaks are shown by the PXRD of DS at 2θ = 13.32°, 24.10°, 29.30° and 35.40°, respectively, as indicated in Figure 5A, which demonstrates the drug crystalline nature. Figure 5B indicates the amorphous network of developed hydrogels. Peaks of unloaded Car934-g-poly (acrylic acid) hydrogels are revealed at 2θ = 15.21°, 21.51°, 28.48° and 37.12°. Similarly, in Figure 5C, peaks of drug loaded hydrogels are observed at 2θ = 13.43°, 19.87°, 26.13° and 31.42°, which are nearly same as those of unloaded hydrogels. The small difference is due to the encapsulation of the drug by the hydrogel network. Likewise, the intensity of all the above-mentioned peaks of the drug are reduced in drug-loaded Car934-g-poly (acrylic acid) hydrogels, which reveal the successful encapsulation of the drug by an amorphous network of hydrogels, as shown in Figure 5C [38]. Naeem, F. et al. also reported the same results, which further supports our study [1].

### 2.5. Dynamic Swelling

#### 2.5.1. Response of pH on Dynamic Swelling

**Figure 6:**
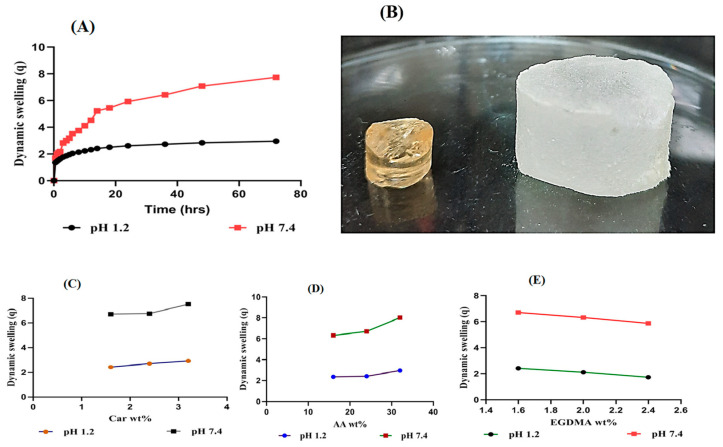
(**A**) Effect of pH on dynamic swelling. (**B**) Dried and swelled forms of the Car934-g-poly(acrylic acid) hydrogels. (**C**) Effects of carbopol, (**D**) Effects of AA and (**E**) EGDMA on the dynamic swelling of the Car934-g-poly(acrylic acid) hydrogels.

The effect of pH on the dynamic swelling of Car934-g-poly (acrylic acid) hydrogels at pH 1.2 and pH 7.4 is shown in Figure 6A,B. pH-dependent swelling is exhibited by all developed Car934-g-poly (acrylic acid) hydrogels, with almost less swelling at pH 1.2, while, at pH 7.4, greater swelling is observed. This absorptive behavior of water of the fabricated hydrogels at pH 1.2 is due to the protonation of –COO groups into –COOH groups in an acidic environment, whereas deprotonation of –COOH groups into –COO groups back occurs in a basic pH environment. Due to the high concentration of –COOH in an acidic medium, Car934-g-poly (acrylic acid) hydrogels are not highly swelled. A high concentration of COOH leads to an increase in hydrogen-bonding interactions among –COOH groups that further increase the compatibility of gels due to a reduction in electrostatic repulsion among these groups, whereas, in a basic medium of pH 7.4, an increase in concentration of –COO occurs, and this further results in (i) an enhancement in electrostatic repulsion among –COO groups and (ii) a reduction in the interaction of hydrogen bonding, thus developing hydrogel swelling more at a high pH (7.4) as compared to a low pH (1.2) (*p* < 0.05) [39]. 

#### 2.5.2. Influence of Carbopol/AA/and Ethylene Glycol Dimethacrylate (EGDMA) on Swelling

The effects of carbopol, AA and EGDMA on the dynamic swelling of Car934-g-poly (acrylic acid) hydrogels are shown in Figure 6C–E. The effect of carbopol on the dynamic swelling of developed hydrogels is higher at a basic medium (pH 7.4) as compared to an acidic medium (pH 1.2). An increase in the dynamic swelling of hydrogels is exhibited at both pH 1.2 and pH 7.4 as the composition of carbopol increases by keeping the composition of the monomer and crosslinker constant (Figure 6C). The swelling of fabricated hydrogels increases as the composition of carbopol increases because of the high concentration of the carboxylic group in carbopol, which ionizes more at a higher pH and leads to repulsion among the same group charges, and as a result, swelling is increased [40].

Similarly, by enhancing the composition of AA, an increase in swelling is detected (Figure 6D) at both pH 1.2 and pH 7.4, respectively. The pKa value of AA is 4.2, so at a low pH, chains of AA are collapsed; due to which, the swelling of the hydrogels at pH 1.2 is less than at pH 7.4. As the pH rises beyond 6 and 8, carboxylate ions are produced by AA, leading to the repulsion of chains and exhibition of higher swelling [41].

Unlikely carbopol and AA shrinkage in the swelling of developed hydrogels is observed at both pH 1.2 and pH 7.4 as the composition of EGDMA is enhanced (Figure 6E). The reason being the highly crosslinked density of the developed hydrogels retards the motility of the network chains, and hence, swelling is decreased [42,43,44]. Another reason is the pore size of the hydrogels network. As the composition of EGDMA increases, the pore size of the developed hydrogels is decreased due to the high gel formation, and as a result, the swelling is decreased and vice versa [45]. 

### 2.6. Sol–Gel Alysis

**Figure 7:**
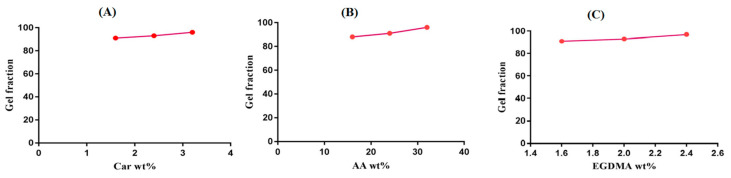
Effects of (**A**) carbopol, (**B**) AA and (**C**) EGDMA on the gel fractions of the Car934-g-poly(acrylic acid) hydrogels.

The effects of the hydrogel contents on the gel fraction are revealed in Figure 7A–C. The gel fraction increases as the concentration of carbopol increases (Figure 7A), while the remaining contents stay constant. The increase in carbopol concentration results in an increase of reactive sites for the polymerization reaction. The higher the carbopol concentration, the higher the reactive sites available for monomer polymerization, and the greater the gel fraction [46]. Similarly, as the concentration of AA increases, an increase in the gel fraction is observed (Figure 7B). Likewise, for carbopol and AA, an increase in the gel fraction is observed as an increase in the concentration of EGDMA occurs (Figure 7C). The higher the EGDMA concentration, the greater the crosslinking density of the polymeric network, and the greater the gel fraction [46,47]. The sol fraction is decreased with an increase in the concentration of the polymer, and the monomer and crosslinker occurs, because the sol fraction is inversely proportional to the gel fraction [48].

### 2.7. Drug Loading Analysis

**Figure 8:**
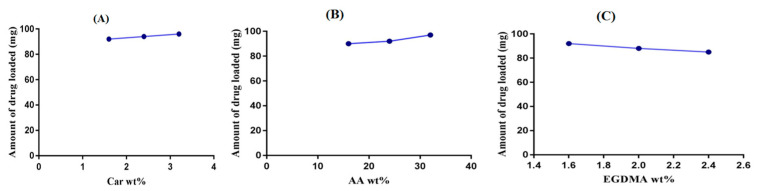
Effects of (**A**) carbopol, (**B**) AA and (**C**) EGDMA on the drug loading of the Car934-g-poly(acrylic acid) hydrogels.

A drug loading analysis was carried out for all developed Car934-g-poly (acrylic acid) hydrogels, as shown in Figure 8A–C. The drug loading increases as the concentration of carbopol increases (Figure 8A). Similarly, by enhancing the concentration of AA, an increase in drug loading is exhibited (Figure 8B). However, contrary to the polymer and monomer, drug loading decreases as the concentration of EGDMA increases (Figure 8C). Drug loading is directly related to the swelling of the hydrogel network; the higher the swelling of the polymeric system, the higher the drug loading and vice versa [49].

### 2.8. In-Vitro Drug Release Studies 

#### 2.8.1. Influence of pH on Drug Release

**Figure 9:**
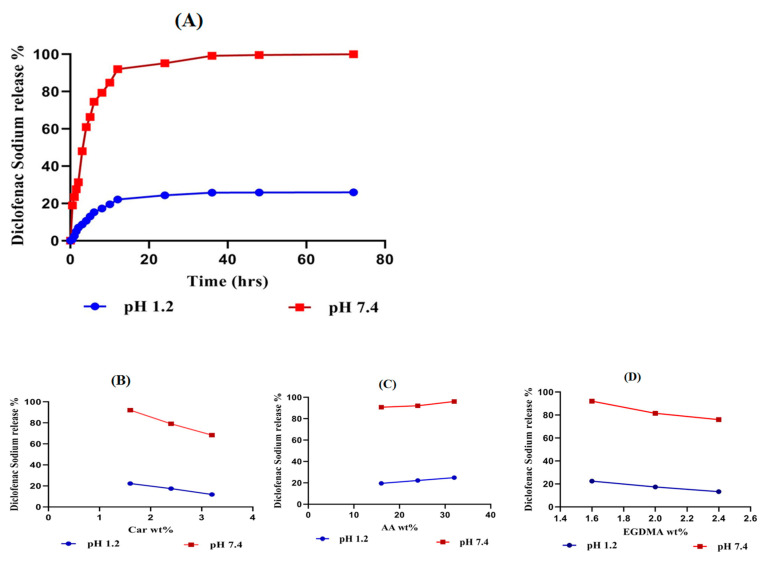
(**A**) Effects of pH on the diclofenac sodium percent release from Car934-g-poly(acrylic acid) hydrogels. (**B**) Effects of carbopol, (**C**) Effects of AA and (**D**) Effects of EGDMA on the diclofenac sodium percent release.

An in-vitro drug release study was conducted for all fabricated Car934-g-poly (acrylic acid) hydrogels at both the acid (pH 1.2) and basic (pH 7.4) mediums, respectively. A high percent release of the drug is observed at pH 7.4 as compared to that at pH 1.2, as shown in Figure 9A. The pH-dependent drug release is due to the pH-dependent swelling of the fabricated hydrogels. Deprotonation of the COOH groups of carbopol and the release of carboxylate ions in excess amounts by AA at higher pH leads to a maximum swelling at pH 7.4, and as a result, a high percent release of drug is observed at pH 7.4; thus, all formulations of the developed hydrogels show a pH-dependent percent drug release [50]. The drug release rate in the pH 7.4 medium is significantly higher (*p* < 0.05) than that in the pH 1.2 medium. 

#### 2.8.2. Influence of Carbopol/AA/and EGDMA on Drug Release

As the concentration of carbopol increases, a slight decline in the percent of drug release from the Car934-g-poly(acrylic acid) hydrogel is observed, as shown in Figure 9B. The reduction in the release of the drug from carbopol is because of the formation of a tight, hard gel. As the drug penetrates the hydrogel network, the viscosity of the system increases, and the drug does not easily come out of the network, so the percent of the drug release decreases as the concentration of carbopol is enhanced [51]. Khan and Zhu et al. also found the same results [52]. Unlike carbopol, the drug release increases as the concentration of AA increases (Figure 9C) while keeping the concentration of other contents constant [53]. Like carbopol, due to the high gel fraction, an increase in EGDMA quantity and decrease in the percent drug release is observed (Figure 9C) [54]. 

### 2.9. Release Kinetic of DS

Different kinetic modeling systems such as zero-order, first-order, Higuchi and Korsmeyer Peppas were applied on all formulations to understand the release order of the drug from the Car934-g-poly (acrylic acid) hydrogels, as shown in Table 1. The best fit model was chosen on the basis of the closeness of the “r” value to 1. For most of the formulations, the values of “r” were found higher for the first-order equation as compared to the zero-order. The zero-order fails to discuss the release order of the drug from the Car934-g-poly (acrylic acid) hydrogels. The “r” values for zero-order range between 0.6878 and 0.9647. Similarly, the “r” values for first-order are obtained in the range of 0.9118–0.9901. A diffusion-controlled release mechanism of the drug was found from the “r” values of Higuchi models. A diffusion-controlled system indicates linearity between the plot of the drug released and a square root of the time for the release. All formulations of the developed hydrogels showed good fit with the Korsmeyer-Peppas model. The “r” values of Korsmeyer-Peppas were higher than the “r” values of the other respective models for most of the developed hydrogel formulations, which indicates that the release order of the drug from the Car934-g-poly acrylic acid) hydrogels exhibits the Korsmeyer-Peppas model. The “*n*” value determines the type of diffusion mechanism, i.e., Fickian diffusion mechanism and non-Fickian diffusion mechanism or anomalous. If the value is *n* = 0.5, it indicates the Fickian diffusion mechanism, whereas, if *n* > 0.5, it means the diffusion is non-Fickian or anomalous. The “*n*” value for all formulations was in the range of 0.5129–0.7413, confirming non-Fickian or anomalous diffusion, as shown in Table 1 [55,56].

## 3. Materials and Methods

### 3.1. Materials

Diclofenac sodium and ethylene glycol dimethacrylate (EGDMA) were purchased from Alfa-Aesar (Ward Hill, MA, USA). Carbopol 934 was obtained from Noveon, Inc (Cleveland, OH, USA). Acrylic acid was procured from Acros (Carlsbad, CA USA). Similarly, ammonium peroxydisulfate was obtained from Showa (Tokyo, Japan).

### 3.2. Methods

#### 3.2.1. Fabrication of Car934-g-poly (acrylic acid) Hydrogels

Carbopol934-graft-poly (acrylic acid) (Car934-g-poly(acrylic acid acid; AA) added dropwise into the polymer solution. The final step was the addition of the required quantity of crosslinker (EGDMA) into the stirred mixture. The resultant solution was then transferred into glass tubes and kept in a water bath at 55 °C for 2 h and 65 °C for 22 h. The cylindrical hydrogels were removed from the glass tubes and cut into pieces of 5-mm and 8-mm thick)) hydrogels were prepared by the free-radical polymerization technique. A specific amount of polymer (Carbopol934) was placed in a beaker, and then, the required amount of deionized distilled water was added and stirred for 20 min at 50 °C temperature. Nitrogen gas was used to purge the polymer solution for 20 min in order to remove any dissolved oxygen. After that, initiator solution ammonium peroxydisulfate (APS) was poured into the solution of the polymer, followed by a monomer solution (acrylic ness, respectively. A mixture of ethanol and water (70:30) was used for washing the prepared gels to remove any unreacted component from the gel. After the initial drying of gel discs for 24 h at room temperature, the discs were then placed in an oven at a temperature of 40 °C for seven days; then, the dried hydrogels discs were used for further studies. The various compositions of the developed hydrogels are given in Table 2. 

#### 3.2.2. FTIR Analysis

FTIR analysis was performed for the model drug, carbopol, acrylic acid, unloaded Carb934-g-poly (acrylic acid) hydrogels and drug-loaded Car934-g-poly (acrylic acid) hydrogels. All the excipients used in the preparation of hydrogels were crushed in a mortar and pestle to the desired size thoroughly and then analyzed by Nicolet 380 FTIR (Thermo Fisher Scientific, MA, USA) in the range of 4000–500 cm^−1^ [21].

#### 3.2.3. Thermal Analysis

Thermogravimetric analysis (TGA) and differential scanning calorimetry (DSC) were performed for DS, carbopol, and Car934-g-poly (acrylic acid) hydrogels while using PerkinElmer Simultaneous Thermal Analyzer STA 8000 and PerkinElmer DSC 4000 (PerkinElmer Ltd., Buckinghamshire, UK) respectively. All samples for thermal analysis were filtered by mesh size 40 to achieve the desired size of the particle after crushing. For the analysis of TGA, a sample quantity was kept in the range of (0.5–5 mg) and then put in an open pan connected to a microbalance. Heat for all samples was maintained at 20 to 400 °C, and dry nitrogen was used throughout the TGA analysis. For the analysis of DSC, precise quantities of the samples were taken in the range of (0.5–3 mg) into an aluminum pan and then examined under nitrogen gas from 50 to 600 °C, with the heating rate kept at 20 °C/min [57].

#### 3.2.4. Scanning Electron Microscopy (SEM) Analysis

SEM SU8010 (Hitachi, Tokyo, Japan) was performed for analysis of the surface morphology of the developed hydrogels. All discs of hydrogel were cut into small slices and fixed on an aluminum stub with double-adhesive tape. Gold was coated on the stubs by a gold sputter under argon atmosphere. By the help of photomicrographs, the surface morphology of all samples was investigated [58]. 

#### 3.2.5. PXRD Analysis

Powder X-ray diffractometry (PXRD) analysis was carried out for DS, unloaded Car934-g-poly (acrylic acid) hydrogels and drug-loaded Car934-g-poly (acrylic acid) hydrogels at 25 °C by using a XRD-6000 (Shimadzu, Kyoto, Japan). A plastic sample holder was used for holding samples, while, for surface leveling, a glass slide was used. For analysis of samples, the theta (θ) was maintained between 10° to 60° at a rate of 2° 2θ/min at 25 °C [59].

#### 3.2.6. Dynamic Swelling Studies

Dynamic swelling studies were carried out to evaluate the pH-sensitive nature of developed hydrogels in both acidic and basic media. Weighed dried hydrogel discs were immersed in the respective swelling media, i.e., pH 1.2 and pH 7.4, respectively. The samples were withdrawn at a specific interval of time, blotted with filter paper to eliminate the spare water and weighed again on a weighing balance [60]. The following given equations were used for calculating dynamic swelling (Equation (1)) and percentage swelling ratio (Equation (2)).

(1) $$\left(q\right)=\frac{{\;W}_{2}}{{\;W}_{1}}$$

where

q = dynamic swelling,
W_1_ = initial weight (before swelling)
and W_2_ = final weight (after swelling) at time t.

(2) $$\left(\mathrm{SR}\%\right)=\frac{{\;M}_{1}-{\;M}_{2}}{M_{2}}\times 100$$

where

M_1_ = weight of swollen hydrogels discs, while M_2_ = weight of dry hydrogel discs.

#### 3.2.7. Determination of Sol–Gel Fraction 

Sol–gel studies were carried out for all fabricated formulations of Car934-g-poly (acrylic acid) hydrogels. The sol portion is the soluble unreacted part of the developed hydrogels, and the Soxhlet extraction method was used for the sol–gel analysis of hydrogels. A specific quantity of hydrogel discs was placed in the Soxhlet apparatus, and the extraction process was continued in deionized boiling water for 9 h. Thereafter, discs of hydrogels were placed in a vacuum oven for 4 days to dry completely, and finally, dried discs of hydrogels were reweighed again for sol–gel determination [60]. The following given equations were used for determination of the sol fraction (Equation (3)) and gel fraction (Equation (4)), respectively.

(3) $$\mathrm{Sol}\;\mathrm{fraction}\;\%=\frac{{\;W}_{1}-{\;W}_{2}}{{\;W}_{2}}\times 100$$

(4) Gel fraction=100−Sol fraction

W1 = initial weight of hydrogels, and W2 = final weight of dried hydrogels.

#### 3.2.8. Drug Loading

The diffusion and absorption method was used for the loading of DS into fabricated hydrogels [61]. Specific weighed quantities of dried hydrogels discs were submerged into 0.8% drug solution for 72 h at 25 °C. After 72 h, the loaded hydrogel discs were washed by distilled water and left at 25 °C for a short interval of time; then, the hydrogel discs were placed at 40 °C in an oven for further drying. Drug loading estimation of loaded hydrogels was analyzed by two methods: (a) the extraction method, where loaded hydrogels discs were submerged in 25-mL fresh phosphate buffer of pH 7.4. This process was continued until all the entrapped drug was released from hydrogel discs. The collected samples were then analyzed on UV–vis spectrophotometer (U-5100, 3J2-0014, Tokyo, Japan) at λ_max_ 260 nm to know the drug contents; and (b) the weight method, which was also used to analyze the quantity of drug loaded in hydrogel discs [62,63], where drug loading of the developed hydrogels was calculated by the given equations (Equations (5) and (6)).

(5) $$\mathrm{Amount}\;\mathrm{of}\;\mathrm{drug}\;=\;W_{D}\;-\;W_{d}$$

While, for the percent of drug loading, the equation is

(6) $$\mathrm{Drug}\;\mathrm{Loading}\;\%\;=\frac{{\;W}_{D}-{\;W}_{d}}{W_{d}}\times 100$$

where WD = weight of dried hydrogel discs after immersion in drug solution, and Wd = weight of dried hydrogel discs before immersion in drug solution.

#### 3.2.9. In-Vitro Study

An in-vitro study was performed for all developed hydrogels at both acidic and basic media, i.e., simulated gastric fluid (pH 1.2) and simulated intestinal fluid (pH 7.4), respectively, in order to know the pH-dependent release of DS from the Car934-g-poly (acrylic acid) hydrogel system. Loaded hydrogel discs were assessed for analysis of DS in 900-mL buffer solutions at both media, i.e., pH 1.2 and 7.4 through a USP Dissolution Apparatus-II at a temperature of 37 °C and 50 rpm. Weighed quantities of loaded hydrogel discs were immersed in 900-mL buffer solutions of both pH 1.2 and 7.4. A sample of 5 mL was withdrawn at a specific time interval, and the same amount of fresh buffer was added to maintain the sink condition. The samples were then analyzed on UV–vis spectrophotometer (U-5100, 3J2-0014, Tokyo, Japan) at 260 nm to evaluate the percent drug release [64].

#### 3.2.10. Release Kinetics of SD

Evaluation of the order and release mechanisms of the drug from the developed hydrogels was carried out by fitting the in-vitro release data of the drug in different kinetic models, such as the zero-order (Equation (7)), first-order (Equation (8)), Higuchi (Equation (9)) and Korsmeyer-Peppas models (Equation (10)) [65].

Zero-order kinetics Ft = K_0_t (7)

where Ft = fraction of drug released at time t, and K_0_ = zero-order release constant.

First-order kinetics ln (1 − F) = K_1_t (8)

where F = fraction of drug released at time t, and K_1_ = first-order release constant.

Higuchi Model F = K_2_t^1/2^ (9)

where F = fraction of drug released at time t, and K_2_ = Higuchi constant.

(10) $$\mathrm{Korsmeyer}\;-\;\mathrm{Peppas}\;\mathrm{Model}\;\frac{Mt}{M\infty }\;=\;K_{3}t^{n}$$

where M_t_ = amount of absorbed water at any time t, M∞ = mass or water uptake at equilibrium, n = drug release exponent and K_3_ = kinetic constant.

#### 3.2.11. Statistical Analysis

Statistical analysis was performed using a computer program, SPSS Statistic software 22.0 (IBM Corp, Armonk, NY, USA). Differences between tests were tested using Student’s *t*-Test and were considered statistically significant if the *p*-value was < 0.05. 

## 4. Conclusions

In the current research work, Car934-g-poly (acrylic acid) hydrogels were prepared successfully by the free-radical polymerization technique while using various compositions of carbopol, acrylic acid and EGDMA. FTIR studies revealed the successful grafting of AA over the carbopol backbone; TGA and DSC showed that prepared hydrogels were thermally stable; SEM indicated that a sporous structure of fabricated hydrogels provided high quantities of water for absorption, while PXRD showed that crystallinity of the drug was reduced by the amorphous network of hydrogels. Higher swelling was observed at pH 7.4 as compared to pH 1.2, and as a result, a high release of the drug was observed at pH 7.4, indicating that developed hydrogel networks exhibit pH-dependent deliveries of water-soluble drugs like diclofenac sodium. Thus, keeping in mind all the above-mentioned studies, Car934-g-poly (acrylic acid) hydrogels are considered to be a suitable drug carrier system for the delivery of aqueous-soluble drugs to a basic medium.

## Figures and Tables

**Table 1:** Kinetic modeling release of diclofenac sodium (DS) from the Car934-g-poly(acrylic acid) hydrogels.

Formulation Code	Zero-Order (r^2^)	First-Order (r^2^)	Higuchi (r^2^)	Korsmeyer-Peppas	
				(r^2^)	*n*
CAAF-1	0.7149	0.9308	0.8949	0.9595	0.5306
CAAF-2	0.8695	0.9631	0.9446	0.9601	0.5135
CAAF-3	0.9647	0.9901	0.9842	0.9838	0.5213
CAAF-4	0.7518	0.9118	0.9009	0.9604	0.5770
CAAF-5	0.7149	0.9308	0.8949	0.9595	0.5306
CAAF-6	0.6878	0.9522	0.8760	0.9424	0.5129
CAAF-7	0.7149	0.9308	0.8949	0.9595	0.5306
CAAF-8	0.9116	0.9669	0.9499	0.9818	0.6605
CAAF-9	0.9151	0.9635	0.9542	0.9877	0.7413

r^2^: regression coefficients; *n*: drug release exponent.

**Table 2:** Feed ratio scheme for the formulation of Car934-g-poly (acrylic acid) hydrogels.

Formulation Code (100 g)	Polymer (Carbopol 934) (g)	Monomer (Acrylic Acid) (g)	Initiator (APS) (g)	Crosslinker (EGDMA) (g)
CAAF-1	1.6	24	0.4	1.6
CAAF-2	2.4	24	0.4	1.6.
CAAF-3	3.2	24	0.4	1.6
CAAF-4	1.6	16	0.4	1.6
CAAF-5	1.6	24	0.4	1.6
CAAF-6	1.6	32	0.4	1.6
CAAF-7	1.6	24	0.4	1.6
CAAF-8	1.6	24	0.4	2.0
CAAF-9	1.6	24	0.4	2.4

One hundred milliliters of aqueous solution of each formulation containing a specific concentration of polymer, monomer and crosslinker. APS: ammonium peroxydisulfate and EGDMA: ethylene glycol dimethacrylate.

## References

[1] Yar M., Shahzad S., Siddiqi S.A., Mahmood N., Rauf A., Anwar M.S., Chaudhry A.A., Rehman I.U. (2015). Triethyl orthoformate mediated a novel crosslinking method for the preparation of hydrogels for tissue engineering applications: Characterization and in vitro cytocompatibility analysis. Mater. Sci. Eng. C.

[2] Samanta H.S., Ray S.K. (2014). Controlled release of tinidazole and theophylline from chitosan based composite hydrogels. Carbohydr. Polym..

[3] Ullah F., Othman M.B.H., Javed F., Ahmad Z., Akil H.M. (2015). Classification, processing and application of hydrogels: A review. Mater. Sci. Eng. C.

[4] Ahmed E.M. (2015). Hydrogel: Preparation, characterization, and applications: A review. J. Adv. Res..

[5] Prabaharan M. (2011). Prospective of guar gum and its derivatives as controlled drug delivery systems. Int. J. Biol. Macromol..

[6] Bhattarai N., Gunn J., Zhang M. (2010). Chitosan-based hydrogels for controlled, localized drug delivery. Adv. Drug Deliv. Rev..

[7] Siepmann J., Siegel R.A., Rathbone M.J. (2012). Fundamentals and Applications of Controlled Release Drug Delivery.

[8] Miyata T., Uragami T., Nakamae K. (2002). Biomolecule-sensitive hydrogels. Adv. Drug Deliv. Rev..

[9] Ulijn R.V., Bibi N., Jayawarna V., Thornton P.D., Todd S., Mart R.J., Smith A.M., Gough J. (2007). Bioresponsive hydrogels. Mater. Today.

[10] Atta S., Khaliq S., Islam A., Javeria I., Jamil T., Athar M.M., Shafiq M.I., Ghaffar A. (2015). Injectable biopolymer based hydrogels for drug delivery applications. Int. J. Biol. Macromol..

[11] Pillai O., Panchagnula R. (2001). Polymers in drug delivery. Curr. Opin. Chem. Biol..

[12] Buenger D., Topuz F., Groll J. (2012). Hydrogels in sensing applications. Prog. Polym. Sci..

[13] Bromberg L. (1998). Temperature-responsive gels and thermogelling polymer matrices for protein and peptide delivery. Adv. Drug Deliv. Rev..

[14] Qiu Y., Park K. (2001). Environment-sensitive hydrogels for drug delivery. Adv. Drug Deliv. Rev..

[15] Bettini R., Colombo P., Peppas N.A. (1995). Solubility Effects on Drug Transport through Ph-Sensitive, Swelling-Controlled Release Systems-Transport of Theophylline and Metoclopramide Monohydrochloride. J. Control. Release.

[16] Fogueri L.R., Singh L.R.F.A.S. (2009). Smart Polymers for Controlled Delivery of Proteins and Peptides: A Review of Patents. Recent Patents Drug Deliv. Formul..

[17] Galaev I. (1999). ’Smart’ polymers and what they could do in biotechnology and medicine. Trends Biotechnol..

[18] Taylor N.W., Bagley E.B. (1977). Tailoring closely packed gel–particle systems for use as thickening agents. J. Appl. Polym. Sci..

[19] Silano V., Baviera J.M.B., Bolognesi C., Brüschweiler B.J., Chesson A., Cocconcelli P.S., Crebelli R., Gott D.M., Grob K., EFSA Panel on Food Contact Materials, Enzymes and Processing Aids (CEP) (2018). Safety assessment of the active substance polyacrylic acid, sodium salt, cross-linked, for use in active food contact materials. EFSA J..

[20] Mun E.A., Hannell C., Rogers S.E., Hole P., Williams A.C., Khutoryanskiy V.V. (2014). On the Role of Specific Interactions in the Diffusion of Nanoparticles in Aqueous Polymer Solutions. Langmuir.

[21] Khalid I., Ahmad M., Minhas M.U., Barkat K. (2018). Preparation and characterization of alginate-PVA-based semi-IPN: Controlled release pH-responsive composites. Polym. Bull..

[22] Liang X.X., Omer A., Hu Z.-H., Wang Y., Yu D., Ouyang X.-K. (2019). Efficient adsorption of diclofenac sodium from aqueous solutions using magnetic amine-functionalized chitosan. Chemosphere.

[23] Altman R., Bosch B., Brune K., Patrignani P., Young C. (2015). Advances in NSAID Development: Evolution of Diclofenac Products Using Pharmaceutical Technology. Drugs.

[24] Agnihotri S.M., Vavia P.R. (2009). Diclofenac-loaded biopolymeric nanosuspensions for ophthalmic application. Nanomed. Nanotechnol. Biol. Med..

[25] Swain R., Nagamani R., Panda S. (2015). Formulation, in vitro Characterization and Stability Studies of Fast Dispersing Tablets of Diclofenac Sodium. J. Appl. Pharm. Sci..

[26] Sahoo S., Chakraborti C.K., Behera P.K., Mishra S.C. (2011). Characterization of mucoadhesive ciprofloxacin suspensions by Fourier transform infrared spectroscopy. Int. J. Pharm. Sci. Rev. Res..

[27] Patel R., Dadhani B., Ladani R., Baria A., Patel J. (2010). Formulation, evaluation and optimization of stomach specific in situ gel of clarithromycin and metronidazole benzoate. Int. J. Drug Deliv..

[28] Moharram M.A., Khafagi M.G. (2007). Application of FTIR spectroscopy for structural characterization of ternary poly(acrylic acid)–metal–poly(vinyl pyrrolidone) complexes. J. Appl. Polym. Sci..

[29] Khalid I., Ahmad M., Minhas M.U., Barkat K., Sohail M. (2016). Cross-Linked Sodium Alginate-g-poly(Acrylic Acid) Structure: A Potential Hydrogel Network for Controlled Delivery of Loxoprofen Sodium. Adv. Polym. Technol..

[30] Naidu V., Madhusudhana K., Sashidhar R., Ramakrishna S., Khar R.K., Ahmed F.J., Diwan P.V. (2009). Polyelectrolyte complexes of gum kondagogu and chitosan, as diclofenac carriers. Carbohydr. Polym..

[31] Loh G.O.K., Tan Y.T.F., Peh K.K. (2014). Hydrophilic polymer solubilization on norfloxacin solubility in preparation of solid dispersion. Powder Technol..

[32] Chun M.-K., Bhusal P., Choi H.-K. (2013). Application of Carbopol/PVP interpolymer complex to prepare mucoadhesive floating granule. Arch. Pharmacal Res..

[33] Shen X., Yu D., Zhu L., Branford-White C., White K., Chatterton N.P. (2011). Electrospun diclofenac sodium loaded Eudragit(R) L 100-55 nanofibers for colon-targeted drug delivery. Int. J. Pharm..

[34] Lee W.F., Chiang W.H. (2004). Swelling and drug-release behavior of the poly(AA-co-N-vinyl pyrrolidone)/chitosan interpenetrating polymer network hydrogels. J. Appl. Polym. Sci..

[35] Shah R., Saha N., Saha P. (2015). Influence of temperature, pH and simulated biological solutions on swelling and structural properties of biomineralized (CaCO3) PVP–CMC hydrogel. Prog. Biomater..

[36] Singh B., Dhiman A. (2019). Functionalization of carbopol with NVP for designing antibiotic drug loaded hydrogel dressings for better wound management. J. Pharm. Biopharm. Res..

[37] Khanum H., Ullah K., Murtaza G., Khan S.A. (2018). Fabrication and in vitro characterization of HPMC-g-poly(AMPS) hydrogels loaded with loxoprofen sodium. Int. J. Biol. Macromol..

[38] Sarfraz R.M., Khan M.U., Mahmood A., Akram M.R., Minhas M.U., Qaisar M.N., Ali M.R., Ahmad H., Zaman M. (2020). Synthesis of co-polymeric network of carbopol-g-methacrylic acid nanogels drug carrier system for gastro-protective delivery of ketoprofen and its evaluation. Polym. Technol. Mater..

[39] Sohail M., Ahmad M., Minhas M.U., Ali L., Munir A., Khalid I. (2014). Synthesis and Characterization of Graft PVA Composites for Controlled Delivery of Valsartan. Lat. Am. J. Pharm..

[40] Sharmin N., Elias-Al-Mamun M., Jalil R.-U. (2010). A Novel Method to Study the Effect of pH and Excipients on Water Uptake and Swelling Behaviour of Carbopol Polymers. Bangl. Pharma. J..

[41] Sullad A.G., Manjeshwar L.S., Aminabhavi T.M. (2010). Novel pH-Sensitive Hydrogels Prepared from the Blends of Poly(vinyl alcohol) with Acrylic Acid-graft-Guar Gum Matrixes for Isoniazid Delivery. Ind. Eng. Chem. Res..

[42] Caykara T., Turan E. (2006). Effect of the amount and type of the crosslinker on the swelling behavior of temperature-sensitive poly(N-tert-butylacrylamide-co-acrylamide) hydrogels. Colloid Polym. Sci..

[43] Teijón C., Olmo R., Blanco M.D., Teijón J.M., Romero A. (2006). Effect of the crosslinking degree and the nickel salt load on the thermal decomposition of poly(2-hydroxyethyl methacrylate) hydrogels and on the metal release from them. J. Colloid Interface Sci..

[44] Teijón J., Trigo R., García O., Blanco M. (1997). Cytarabine trapping in poly(2-hydroxyethyl methacrylate) hydrogels: Drug delivery studies. Biomaterials.

[45] Vazquez B., Gurruchaga M., Goni I. (1995). Hydrogels Based on Graft-Copolymerization of Hema Bma Mixtures onto Soluble Gelatin-Swelling Behavior. Polymer.

[46] Harish N.M., Prabhu P., Charyulu R.N., Gulzar M.A., Subrahmanyam E.V.S. (2009). Formulation and evaluation ofin situgels containing clotrimazole for oral candidiasis. Indian J. Pharm. Sci..

[47] Hussain T., Ranjha N.M., Shahzad Y. (2011). Swelling and Controlled Release of Tramadol Hydrochloride from a pH-Sensitive Hydrogel. Des. Monomers Polym..

[48] Dergunov S.A., Nam I.K., Mun G.A., Nurkeeva Z.S., Shaikhutdinov E.M. (2005). Radiation synthesis and characterization of stimuli-sensitive chitosan–polyvinyl pyrrolidone hydrogels. Radiat. Phys. Chem..

[49] Murthy P.K., Mohan Y.M., Sreeramulu J., Raju K.M. (2006). Semi-IPNs of starch and poly(acrylamide-co-sodium methacrylate): Preparation, swelling and diffusion characteristics evaluation. React. Funct. Polym..

[50] Rashid H., Ahmad M., Minhas M.U., Sohail M., Aamir M.F. (2015). Synthesis and Characterization of Poly(hydroxyethyl methacrylate-co-methacrylic acid) Cross Linked Polymeric Network for the Delivery of Analgesic Agent. J. Chem. Soc. Pak..

[51] Khan G.M., Jiabi Z. (1998). Formulation and in vitro evaluation of ibuprofen-carbopol^®^ 974P-NF controlled release matrix tablets III: Influence of co-excipients on release rate of the drug. J. Control. Release.

[52] Khan G.M. (1999). Studies on drug release kinetics from ibuprofen-carbomer hydrophilic matrix tablets: Influence of co-excipients on release rate of the drug. J. Control. Release.

[53] Sanli O., Ay N., Isiklan N. (2007). Release characteristics of diclofenac sodium from poly(vinyl alcohol)/sodium alginate and poly(vinyl alcohol)-grafted-poly(acrylamide)/sodium alginate blend beads. Eur. J. Pharm. Biopharm..

[54] Akhtar M.F., Ranjha N.M., Hanif M. (2015). Effect of ethylene glycol dimethacrylate on swelling and on metformin hydrochloride release behavior of chemically crosslinked pH–sensitive acrylic acid–polyvinyl alcohol hydrogel. DARU J. Pharm. Sci..

[55] Maziad N.A., El-Hanouly S., Zied E., El Kelani T.A., Nasef N.R. (2015). Radiation preparation of smart hydrogel has antimicrobial properties for controlled release of ciprofloxacin in drug delivery systems. Asian J. Pharm. Clin. Res..

[56] Shoaib M.H., Tazeen J., Berardi A., Yousuf R.I. (2006). Evaluation of drug release kinetics from ibuprofen matrix tablets using HPMC. Pak. J. Pharm. Sci..

[57] Mahmood A., Ahmad M., Sarfraz R.M., Minhas M.U., Yaqoob A. (2016). Formulation and in Vitro Evaluation of Acyclovir Loaded Polymeric Microparticles: A Solubility Enhancement Study. Acta Pol. Pharm.-Drug Res..

[58] Sarfraz R.M., Khan H.U., Mahmood A., Ahmad M., Maheen S., Sher M. (2015). Formulation and Evaluation of Mouth Disintegrating Tablets of Atenolol and Atorvastatin. Indian J. Pharm. Sci..

[59] Mahmood A., Ahmad M., Sarfraz R.M., Minhas M.U. (2016). Development of Acyclovir Loaded β-Cyclodextrin-g-Poly Methacrylic Acid Hydrogel Microparticles: An In Vitro Characterization. Adv. Polym. Technol..

[60] Singh B., Sharma N. (2009). Mechanistic Implication for Cross-Linking in Sterculia-Based Hydrogels and Their Use in GIT Drug Delivery. Biomacromolecules.

[61] Sohail M., Ahmad M., Minhas M.U., Ali L., Khalid I., Rashid H. (2015). Controlled delivery of valsartan by cross-linked polymeric matrices: Synthesis, in vitro and in vivo evaluation. Int. J. Pharm..

[62] Kheradmandnia S., Vasheghani-Farahani E., Nosrati M., Atyabi F. (2010). Preparation and characterization of ketoprofen-loaded solid lipid nanoparticles made from beeswax and carnauba wax. Nanomed. Nanotechnol. Biol. Med..

[63] Bukhari S.M.H., Khan S., Rehanullah M., Ranjha N.M. (2015). Synthesis and Characterization of Chemically Cross-Linked Acrylic Acid/Gelatin Hydrogels: Effect of pH and Composition on Swelling and Drug Release. Int. J. Polym. Sci..

[64] Yu J., Warnke J., Lyubchenko Y.L. (2011). Nanoprobing of α-synuclein misfolding and aggregation with atomic force microscopy. Nanomed. Nanotechnol. Biol. Med..

[65] Peppas N.A., Sahlin J.J. (1989). A simple equation for the description of solute release. III. Coupling of diffusion and relaxation. Int. J. Pharm..

